# Impact of patient characteristics, education and knowledge on emergency room visits in patients with asthma and COPD: a descriptive and correlative study

**DOI:** 10.1186/1471-2466-9-43

**Published:** 2009-09-07

**Authors:** Margareta Emtner, Anna Hedin, Mikael Andersson, Christer Janson

**Affiliations:** 1 Department of Neuroscience, Physiotherapy, Uppsala University, SE-751 85 Uppsala, Sweden; 2 Department of Medical Sciences, Respiratory Medicine and Allergology, Uppsala University, SE-751 85 Uppsala, Sweden; 3 Unit for Development of Teaching and Learning, Uppsala University, SE-751 05 Uppsala, Sweden; 4 Physiotherapy Section, Akademiska sjukhuset, SE-751 85 Uppsala, Sweden

## Abstract

**Background:**

Asthma and COPD are major health problems and an extensive burden for the patient and the health care system. Patient education has been recommended, but the influence on knowledge and health outcomes is not fully examined. Our aims were to compare patient characteristics, education and knowledge in patients who had an emergency room (ER) visit, to explore factors related to disease knowledge, and to investigate patient characteristics, patient education and knowledge in relation to further ER visits over a 12 month period.

**Methods:**

Eighty-four patients with asthma and 52 with COPD, who had had an ER visit, were included. They were interviewed by telephone 4 to 6 weeks after the ER visit and followed for a year.

**Results:**

Patients with COPD were older, more sedentary, had had more ER visits the previous year, and had more co morbidity than patients with asthma. About 80% of the patients had received information from health professionals or participated in education/rehabilitation, but a minority (< 20%) reported that their knowledge about how to handle the disease was good. Patients with "good knowledge" were younger, were more likely to have asthma diagnose, and had a higher educational background (p < 0.05). Sixty-seven percent of the patients with COPD had repeated ER visits during the following year versus 42% in asthma (p < 0.05) (adjusted HRR: 1.73 (1.03-2.90)). Patients who had had ER visits the year before inclusion had a higher risk of ER visits the following year (adjusted HRR: 3.83 (1.99-7.38)). There were no significant differences regarding patient education and knowledge between the group with and without further ER visits after adjusting for sex, diagnose, age, and educational background.

**Conclusion:**

Patients with asthma had a better self reported knowledge of disease management and were less likely to have new exacerbations than patients with COPD. Reported level of knowledge was, however, in it self not a predictor of exacerbations. This indicates that information is not sufficient to reduce the burden of disease. Patient education focused on self-management and behavioral change should be emphasized.

## Background

Asthma and chronic obstructive pulmonary disease (COPD) are major health problems and an extensive burden on the patient, health care system, and the economy [1]. The prevalence of asthma has increased over the last 20 years [2], and the prevalence of COPD is 9 to 10% in individuals ≥ 40 years [3]. In asthma, patients with a poor asthma control [4] and persistent asthma are associated with higher costs [5]. Hospitalisation and emergency room (ER) visits account for about 50% of the total costs [6]. Also in COPD the costs are extensive as over 60% are readmitted to hospital within a year after a hospital admission [7].

It has previously been shown that exacerbations in asthma are associated with psychological dysfunction [8,9], poor symptom and disease control [10-12], older age [4], high doses of inhaled corticosteroids (ICS), oral corticosteroids, concomitant chronic sinusititis, and having a long history of asthma [13]. In COPD, exacerbations are associated with impaired quality of life [14,15], increased mortality [16,17], limitations of daily activities [18], disease progression [19,20], poor lung function, previous admissions, under prescription of oxygen [21], increased risk of readmission [7], and low physical capacity [22].

A key component of management guidelines in asthma [2] and COPD [23], is the recommendation for patient education. In asthma, education (information only) improved patient knowledge, but did not have an impact on health outcomes [24], whereas education programs including self-management strategies, i.e. aiming at life-style change, could reduce hospitalisation and ER visits [25]. In COPD, a Cochrane review found that self-management education had no effect on hospital admissions, ER visits, days lost from work, and lung function [26], whereas a Canadian study reported that hospitalisations and ER visits could be reduced with self-management strategies [27,28].

Although disease severity and psycho-social factors are well known contributors to asthma and COPD morbidity, the influence of education and patient knowledge have received less attention. There is little work in routine practice setting which prospectively examines the relationship of patient education and knowledge, and the serious events of repeated ER visits.

The first aim was to compare patient characteristics, education and knowledge in patients with asthma and COPD, who had an ER visit because of an exacerbation. The second aim was to explore factors related to disease knowledge, and the third aim was to compare patients with further ER visits for breathing problems over a 12 month period versus patients with no further ER visits in relation to patient characteristics, patient education and knowledge.

## Methods

### Study design

This was a prospective study of patients with asthma or COPD, who had ER visit because of exacerbation. The University hospital in Uppsala, Sweden, one local hospital, and four general practitioners in the area in and around Uppsala, took part in the study. The study was approved by the ethical committee, Uppsala University. Informed consent was obtained from the patients.

### Subjects

One hundred and sixty-one consecutive patients ≥ 18 years with exacerbation of obstructive lung disease, who had ER visits, and had a previous physician diagnosed asthma or COPD, according to the medical record (J 44 or J 45 (ICD-10)), were during their ER visit invited to participate in the study. Only one patient declined to participate. Four to six weeks later a research nurse called the patients to ask if they still wanted to take part in the study. Two patients had died, three had severe lung cancer diagnose, and 19 patients were not willing to participate or were impossible to get in contact with. Thus, 136 patients were finally included in the study. All 136 patients were followed up at six and 12 months by a research nurse, who called each patient to ask for ER visits. She also checked the patient-reported ER visits in medical records mainly by calling hospitals. All records were reviewed by the investigator to confirm the diagnosis.

### Measurements

The following data were collected four to six weeks after the ER visit:

#### Structured telephone interview

Demographic characteristics, smoking history, level of formal education, level of physical activity, employment status, housing situation, medication, co morbidity, prior ER visits and hospitalizations, and whether they had received patient education (individual or in a group setting), pulmonary rehabilitation and/or written action plan. Three questions were included to identify personal perceptions of current knowledge; knowledge about what can cause an exacerbation, knowledge about what happens in your body during an exacerbation, and knowledge about how to act when getting an exacerbation (Additional file 1: Table S1). Patients scored their knowledge on a four-graded scale (good knowledge, some knowledge, little knowledge, no knowledge).

#### Follow up

Enrolled patients were contacted by phone six and 12 months after inclusion in order to obtain information regarding number of ER visits and hospitalizations. Data was confirmed by checking hospital records.

### Statistics

The Chi-squared test and an unpaired t-test were used when comparing patients with asthma to patients with COPD, and when comparing patients that had or had not had additional ER visits after inclusion to the study. Mann-Whitney's U-test was used when comparing patients with "good knowledge" versus "some, little or no knowledge". The time until the following ER visit was analysed by the Kaplan-Meier survival analysis and Cox regression. The Cox regression model was used to calculate adjusted hazard ratios. The hazard ratios were adjusted for sex, diagnose, age and educational level but only one knowledge related variable was included in each model in order to avoid collinearity. A p-value of < 0.05 was considered statistically significant.

## Results

### Subjects

Eighty-four patients with asthma and 52 patients with COPD, who had had an ER visit because of an exacerbation, were included and followed for a year (Table 1). Patients with COPD were significantly older, more sedentary, ex-smokers, had a lower educational background, more co morbidity, and had had more ER visits the previous year compared to patients with asthma. Both groups of patients were characterized by having had respiratory symptoms for many years, often having had ER visits the year before and being physically inactive. The majority of patients used inhaled corticosteroids (ICS) and/or bronchodilators.

**Table 1:** Patient characteristics in the asthma and COPD groups, mean ± SD and %.

	**Asthma** **n = 84**	**COPD** **n = 52**	**p-value**
Age, years	55 ± 18	69 ± 9	< 0.01

Sex, % female	64	50	0.12

BMI, kg/m^2^	26 ± 5	26 ± 6	0.47

Respiratory symptoms, years	18 ± 14	15 ± 13	0.26

Ever smoked, %	52	94	< 0.01

Pack years, years	8 ± 14	32 ± 21	< 0.01

Current smokers, %	18	19	0.84

ER visits the previous year, %	61	81	0.02

High school or university education (>10 years), %	62	27	< 0.01

Physical activity, mainly sitting, %	10	37	< 0.01

Physical activity, sitting and walking < 4 days per week, %	93	96	0.45

LABA, %	59	64	0.59

ICS, %	90	76	0.03

LABA + ICS, %	54	60	0.53

Tiotropium, %	3	28	0.01

Co-morbidity, %	34	51	< 0.05

ER: Emergency room visits, BMI: Body Mass Index; LABA: Long Acting Beta Agonist; ICS: Inhaled Corticosteroid

Most patients, independent of diagnose, had received information regarding their disease from a physician or nurse, but few had participated in more formal education (group education) or rehabilitation (education and physical training) (Table 2). While patients with asthma seem to receive better education about the disease than patients with COPD (64% vs. 46%, respectively), the asthma group still wanted to learn more about handling the disease (41%) than those with COPD (33%).

**Table 2:** Subjective experience on received patient education in the asthma and COPD groups, %.

	**Asthma** **n = 84**	**COPD** **n = 52**	**p-value**
***Education***			

Information about medications	86	79	0.18

Information about medications by physician	68	60	0.37

Information about medications by nurse	20	17	0.68

Information about what can cause an exacerbation	64	46	0.04

Information about what happens in your body during an exacerbation	57	25	< 0.01

Information on how to act when getting an exacerbation	68	56	0.16

Participation in group education	17	23	0.25

Participation in rehabilitation	5	17	0.02

Written plan	16	14	0.75

***Need for more knowledge***			

Wants to learn more in general	77	71	0.23

Wants to learn more about medications	33	27	0.46

Wants to learn more about the disease	38	39	0.92

Wants to learn how to handle exacerbations	41	33	0.36

Wants a written plan	27	21	0.50

Group education = theoretical education in a group of asthma or COPD patients

Rehabilitation = education and physical training

All patients who had participated in rehabilitation are also included in group education

### Patient education and factors associated with good knowledge

Though many patients had received information, few patients (< 20%) reported that their knowledge was "good" (Additional file 1: Table S1). Patients with "good knowledge" were younger, had had respiratory symptoms for more years, were more likely to have asthma diagnosis, and had a higher educational background.

### Characteristics of patients with further ER visits during the following year

**Figure 1:**
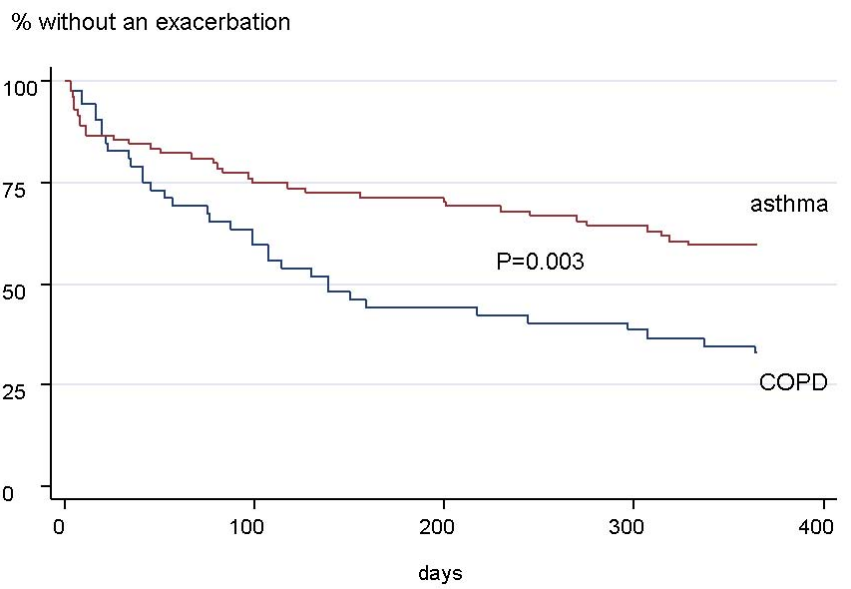
**Patients who performed ER visits within the following year**. Patients with further ER visits were more likely to have COPD (67 vs. 42%, p < 0.05).

Fifty-two percent had at least one ER visit because of exacerbation of obstructive lung disease within the following year. Patients with further ER visits were more likely to have COPD (67 vs. 42%, p < 0.05) (Figure 1). The adjusted hazard ratio, HRR (95% CI) was 1.73 (1.03-2.90). In addition, patients who had had ER visits the year before inclusion had a higher risk of ER visits the following year, adjusted HRR was 3.83 (1.99-7.38). Also patients with hospital admissions because of exacerbation the year before inclusion had a higher risk of ER visits, adjusted HRR was 2.31 (1.29-4.12).

Regarding patient education and knowledge there were no significant differences between the group with and without further ER visits after adjusting for sex, diagnose, age, and educational background (Table 3). The majority in both groups wanted to learn more and about 20% wanted a written action plan.

**Table 3:** Patient education and knowledge.

	**No further emergency room visit** **n = 66**	**At least one emergency room visit** **n = 70**	**HRR**
***Patient education***			

Information about medications	83	85	1.37 (0.67-2.80)

Information about medications by physician	59	70	1.69 (0.97-2.95)

Information about medications by nurse	19	19	1.06 (0.56-2.00)

Information about what can cause an exacerbation	65	50	0.75 (0.46-1.24)

Information about what happens in your body during an exacerbation	47	43	1.28 (0.74-2.21)

Information on how to act when getting an exacerbation	64	63	1.21 (0.73-2.00)

Written plan	12	17	1.47 (0.78-2.76)

***Patient knowledge***			

Good knowledge about what can cause an exacerbation	21	13	0.87 (0.41-1.83)

Good knowledge about what happens in your body during an exacerbation	15	13	1.34 (0.63-2.78)

Good knowledge about what to do when getting an exacerbation	48	43	1.48 (0.71-3.08)

***Need for more knowledge***			

Wants to learn more in general	70	80	1.09 (0.68-1.74)

Wants a written plan	26	23	1.27 (0.66-2.44)

Patient education and knowledge in the groups with no further emergency room visits versus at least one emergency room visit during the following year, numbers in percent and HRR. Adjusted for sex, diagnose, age/10 years, and educational level (>10 years).

## Discussion

This study has shown that patients with COPD and an acute ER visit are more sedentary, have more co morbidity, and had had more ER visits the previous year compared to patients with asthma. Most patients had received information, but a minority had good knowledge about the disease. Patients with "good knowledge" were younger, were more likely to have asthma diagnose, and had a higher educational background. COPD patients were also more likely to have repeated ER visits during the follow-up than patients with asthma. There were no significant differences regarding patient education and knowledge between the group with and without further ER visits after adjusting for sex, diagnose, age, and educational background.

The purpose of this study was to investigate patient characteristics, patient education and knowledge in relation to ER visits in order to identify factors that might help us to get a better understanding of which patients and why patients continued to visit the ER. With a better understanding we might be able to tailor patient education and treatment more specifically and individually. Although many studies have investigated the effects of patient education and rehabilitation in patients with asthma [24,25,29], and COPD [29], no study has investigated to what extent patients with asthma or COPD are informed/taught about the disease and whether there is an association between patient information/education, patient knowledge and ER visits.

### Patient education and knowledge

Patient education has been emphasized in guidelines to be included in the treatment of patients with asthma [2] or COPD [30,31], and there is a general agreement that patient education improves patient knowledge, but the impact on health outcomes is less well established. In this study, where patient education mainly consisted of information about medications and how to act when getting an exacerbation, patients' subjective experience of their knowledge was poor, and many continued to do ER visits [24,26,32]. Though patients in the telephone interview were asked about type of patient education, we were not able (from the interview) to fully identify whether the education consisted of advice, counselling, self-management strategies or behavioural intervention. However, when contacting the included hospitals and general practitioners, we could identify that almost all patients had only received information/advice.

In contrast, self-management programs and sustained patient education in patients with asthma have proven to be successful in improving quality of life and in reducing the economic burden of disease [25,33,34]. Patients with acute asthma who took part in a self-management program twice for 30 minutes and were given a written self-management plan were less likely to be readmitted during a 12-month follow-up period than those without a self-management plan [35]. Adult patients with asthma who had taken part in an educational program for one year had significantly fewer ER visits and hospital admissions after 3 years compared to a control group [36]. In COPD, self-management programs resulted in positive effects on the patients' daily life and wellbeing [26], reduction of exacerbations [37], reduced ER visits [27,28,38], and hospital admissions [39]. COPD patients who had taken part in a disease-specific self-management multi-component program of skill-oriented teaching reduced ER visits up to two years after the program [28].

Thus, it seems that the way patient education is performed is associated with the outcome. Patients included in our study had mainly received information about medications which might explain their poor knowledge in handling the disease and the great number of repeated ER visits. Instead patient education should aim at modifying the behaviour of patients by improving self-management skills [2,23,30,40]. Behavioural research suggests that patient education should focus on attitude, social support and self-efficacy in order to modify behavioural patterns and coping style [41].

### Repeated emergency room visits

Sixty-seven percent of the COPD patients in this study had repeated ER visits during the following year, which is in accordance with other studies [7,42,43]. In Spain, 63% were readmitted within the following year [7], and in the Nordic countries 61% [42]. In asthma, 42% had repeated ER visits, which is higher than have been reported in several previous studies [10,12]. This may be explained by our study group, which was recruited during an ER visit, while patients with asthma in other studies were recruited when attending hospital or primary care for a scheduled visit [10,12].

In this study, significantly more patients with an increased risk of repeated ER visits had had ER visits or had had hospital admissions the year before inclusion, which is in accordance with other investigators studying patients with asthma [10,35,44] or COPD [17,21,43]. Compared to results from other investigators [10], we couldn't find a significant difference between patients who had a written action plan (15% had a written plan) or who used regular corticosteroids in regard to ER visits the following year. Though we didn't measure disease severity, our patients may have had a moderate or severe disease, as all of them had at least one ER visit and 90% of the asthma patients used ICS. As the level of physical activity was extremely low in all our patients a comparison between physically active versus inactive patients was not possible. However, it has been shown that patients with COPD who had an ER visit because of an exacerbation were extremely physically inactive on weight-bearing activities (walking and standing) during hospitalization [45]. The low physical activity level remained one month after discharge and was lower compared to COPD patients without a recent exacerbation. In addition, patients with hospitalization for an exacerbation within the previous year had an even lower activity level. Also in patients with asthma the physical activity level is low [46], and only about 25% of subjects with asthma in the US were considered to be active [47]. Thus, neither patients with COPD nor asthma meet the current recommendations for physical activity [48]. These data are in accordance with ours and are important to highlight as the adverse health effects of inactivity are tremendous.

We had expected a greater proportion of patients to have COPD, as the currently available pharmacotherapy is theoretically more efficient in achieving asthma control than in controlling COPD [49]. However, despite the availability of highly effective drugs research has shown that the control from asthma is far from optimal [12].

### Limitations of the study

Most patients in our study had only received information, thus we could not compare different educational components, and we could not identify if information/advice is sufficient for some patients, i.e. with good educational background. Patients with milder diseases were probably underrepresented since the patients were recruited at an ER setting.

Lung function was not measured, which would have been valuable in patients with COPD [21], but of less importance in patients with asthma [24] in order to identify the risk of ER visits. In our study, the previous ICD-10 diagnosis was used and a lung function test would have been valuable in order to confirm the diagnosis. Unfortunately systemic inflammation was not measured as it has been shown that COPD patients with a heightened systemic inflammation are at increased risk of frequent exacerbations [50].

## Conclusion

Patients with asthma had a better self reported knowledge of disease management and were less likely to have new exacerbations than patients with COPD. Reported level of knowledge was, however, in itself not a predictor of exacerbations. This indicates that information is not sufficient to reduce the burden of disease. Patient education focused on self-management and behavioral change should be emphasized.

## Competing interests

The authors declare that they have no competing interests.

## Authors' contributions

ME designed the study, coordinated the data collection, analyzed data, and wrote the paper. AH designed the study and contributed with important comments during the analysis and writing process. MA analyzed data and contributed with important comments during the analysis. CJ designed the study, contributed with important comments during the statistical analysis, and during the writing process. All authors read and approved the final manuscript.

## Pre-publication history

The pre-publication history for this paper can be accessed here:



## Supplementary Material

Additional file 1 **Table S1. Subjective knowledge, mean ± SD and %**. The data provided describe three questions to identify personal perceptions of current knowledge; knowledge about what can cause an exacerbation, knowledge about what happens in your body during an exacerbation, and knowledge about how to act when getting an exacerbation. Patients scored their knowledge on a four-graded scale (good knowledge, some knowledge, little knowledge, no knowledge). Click here for file
