# Supplementary material for: Impact of patient characteristics, education and knowledge on emergency room visits in patients with asthma and COPD: a descriptive and correlative study
Source: BMC Pulm Med. 2009 Sep 7;9:43. doi: 10.1186/1471-2466-9-43 (PMC2751756; doi:10.1186/1471-2466-9-43)
Supplement: Additional file 1 — Table S1. Subjective knowledge, mean ± SD and %. The data provided describe three questions to identify personal perceptions of current knowledge; knowledge about what can cause an exacerbation, knowledge about what happens in your body during an exacerbation, and knowledge about how to act when getting an exacerbation. Patients scored their knowledge on a four-graded scale (good knowledge, some knowledge, little knowledge, no knowledge). [file 1471-2466-9-43-S1.doc]

Table 1. Subjective knowledge, mean ± SD and %.

|  | What can cause an exacerbation? | | | What happens in your body during an exacerbation? | | | How should you act when getting an exacerbation? | | |
| --- | --- | --- | --- | --- | --- | --- | --- | --- | --- |
|  | Good knowledge  n =19 | Some, little or no knowledge  n =117 | p-value | Good knowledge  n = 23 | Some, little or no knowledge  n = 113 | p-value | Good knowledge  n = 17 | Some, little or no knowledge  n = 119 | p-value |
| Age, years | 51±14 | 62±16 | <0.01 | 51±17 | 62±16 | <0.01 | 52±14 | 61±17 | 0.02 |
| Number of years with the disease, years | 22±11 | 15±14 | <0.01 | 23±11 | 15±14 | <0.01 | 24±14 | 15±13 | <0.01 |
| Asthma diagnose, % | 87 | 57 | <0.01 | 84 | 58 | 0.03 | 82 | 59 | 0.06 |
| High school or university education  (>10 years), % | 39 | 19 | 0.02 | 42 | 19 | 0.02 | 35 | 20 | 0.16 |
| Received information, % | 96 | 50 | <0.000 | 89 | 38 | <0.000 | 100 | 58 | <0.000 |

The data provided describe three questions to identify personal perceptions of current knowledge; knowledge about what can cause an exacerbation, knowledge about what happens in your body during an exacerbation, and knowledge about how to act when having an exacerbation. Patients scored their knowledge on a four-graded scale (good knowledge, some knowledge, little knowledge, no knowledge).
